# Crosstalk between beta‐adrenergic and insulin signaling mediates mechanistic target of rapamycin hyperactivation in liver of high‐fat diet‐fed male mice

**DOI:** 10.14814/phy2.14958

**Published:** 2021-07-07

**Authors:** Sadia Ashraf, Nadia Ashraf, Gizem Yilmaz, Romain Harmancey

**Affiliations:** ^1^ Department of Physiology and Biophysics University of Mississippi Medical Center Jackson MS USA; ^2^ Mississippi Center for Obesity Research University of Mississippi Medical Center Jackson MS USA; ^3^ Jinnah Hospital Lahore Punjab Pakistan

**Keywords:** fatty liver, glycogen, high‐fat diet, insulin, sympathetic nervous system

## Abstract

Nonalcoholic fatty liver disease (NAFLD) is the most common cause of chronic liver disease. While increased nutrient intake and sympathetic activity have been associated with the disease, the pathogenesis of NAFLD remains incompletely understood. We investigated the impact of the interaction of high dietary fat and sugar intake with increased beta‐adrenergic receptor (β‐AR) signaling on the activity of nutrient‐sensing pathways and fuel storage in the liver. C57BL/6J mice were fed a standard rodent diet (STD), a high‐fat diet (HFD), a high‐fat/high‐sugar Western diet (WD), a high‐sugar diet with mixed carbohydrates (HCD), or a high‐sucrose diet (HSD). After 6 week on diets, mice were treated with isoproterenol (ISO) and the activity of liver mTOR complex 1 (mTORC1)‐related signaling analyzed by immunoblotting and correlated with tissue triglyceride and glycogen contents. ISO‐stimulated AKT‐ and ERK‐mediated activation of mTORC1 in STD‐fed mice. Consumption of all four high‐calorie diets exacerbated downstream activation of ribosomal protein S6 kinase beta‐1 (S6K1) in response to ISO. S6K1 activity was greater with the fat‐enriched HFD and WD and correlated with the presence of metabolic syndrome and a stronger activation of AKT and ERK1/2 pathways. Fat‐enriched diets also increased triglyceride accumulation and inhibited glycogen mobilization under β‐AR stimulation. In conclusion, crosstalk between β‐AR and insulin signaling may contribute to HFD‐induced hepatic steatosis through ERK1/2‐ and AKT‐mediated hyperactivation of the mTORC1/S6K1 axis. The findings provide further rationale for the development of therapies aimed at targeting augmented β‐AR signaling in the pathogenesis of NAFLD.

## Graphical abstract

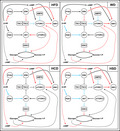

**New & Noteworthy**

We show here that high‐calorie diets exacerbate liver ribosomal protein S6 kinase beta‐1 activation in response to isoproterenol, and that high‐fat diets act partly through enhancement of AKT and ERK signaling, leading to inhibition of glycogenolysis and greater hepatic steatosis. Altogether, the findings expand our knowledge on the mechanisms by which increased sympathetic nervous activity may contribute to the pathogenesis of nonalcoholic fatty liver disease.

## 1 INTRODUCTION

With an incidence hovering over 25% in many countries, nonalcoholic fatty liver disease (NAFLD) has become the most common cause of chronic liver disease worldwide (Fan et al., 2017; Vernon et al., 2011). NAFLD is primarily characterized by an aberrant accumulation of triglycerides in hepatocytes that most frequently results from the impact of obesity, type 2 diabetes, and the metabolic syndrome on liver metabolism (Angulo, 2007). An excess supply of dietary lipids and carbohydrates and the upregulation of de novo lipogenesis are well‐established contributors to hepatic steatosis (Softic et al., 2016). Indeed, consumption of high‐calorie diets enriched predominantly in fat or sugars, or with both macronutrients together, has been linked to NAFLD development in patients and experimental rodents (Clapper et al., 2013; Fernandes‐Lima et al., 2015; Parry & Hodson, 2017; Pompili et al., 2020; Velazquez et al., 2019). Although initially asymptomatic, NAFLD can progress to the more severe form of nonalcoholic steatohepatitis, which can then further deteriorate into cirrhosis and hepatocellular carcinoma (Angulo, 2007). Because long‐term dietary and lifestyle changes are rarely sustainable, NAFLD is on track to become the most common indicator for liver transplant within the next 20 years (Benedict & Zhang, 2017). Therefore, a better understanding of the molecular signaling mechanisms linking excess dietary intake of fat and carbohydrates to the pathogenesis of NAFLD could be the key to designing more effective prevention strategies.

Mechanistic target of rapamycin (mTOR) is one of the core components of the two distinct protein complexes mTORC1 and mTORC2. Both complexes integrate nutrient and hormonal signals for the stimulation of cell growth, survival, and proliferation while simultaneously maintaining energy homeostasis (Saxton & Sabatini, 2017; Yuan et al., 2013). In the liver, mTOR has been implicated in the regulation of ketogenesis, lipogenesis, and more recently glycogen metabolism (Kucejova et al., 2016; Lamming & Sabatini, 2013). Both insulin and glucose promote mTORC1 activation through the induction or inhibition of phosphorylation events orchestrated by AKT, ERKs, and AMP‐activated protein kinase (AMPK; Saxton & Sabatini, 2017; Yuan et al., 2013). Cell‐based studies have implicated activation of the mTORC1/p70S6 kinase 1 (S6K1) signaling axis in the upregulation of sterol regulatory element‐binding proteins (SREBPs) and induction of de novo lipid synthesis downstream of AKT (Duvel et al., 2010; Porstmann et al., 2008). Consequently, hyperactivation of mTORC1 resulting from excess nutrient supply and selective insulin resistance has been proposed as a central mechanism in the pathogenesis of NAFLD (Bar‐Tana, 2020; Kubrusly et al., 2010). However, this hypothesis has been challenged by the fact that mice with constitutive hepatic mTORC1 activity fail to develop NAFLD. In those animals, resistance to diet‐induced hepatic steatosis seems to be due at least in part to an increased feedback inhibition of insulin signaling at the level of the insulin receptor substrate 1 through S6K1‐dependent and ‐independent mechanisms (Kenerson et al., 2015; Kucejova et al., 2016; Yecies et al., 2011). While such mechanism would go against the current paradigm of selective insulin resistance, the potential involvement of alternate hormonal signals in promoting hepatic mTORC1 hyperactivation through AKT under excess nutrient supply remains largely explored.

Adrenergic signaling is part of the sympathetic nervous system and is activated upon stimulation by the catecholamines epinephrine and norepinephrine. Although hepatic nerves have a limited role in the homeostatic control of liver metabolism, adrenergic signaling may be involved in the pathogenesis of NAFLD (Amir et al., 2020; Puschel, 2004). Thus, overexpression of β1‐ and β2‐adrenergic receptor (AR) subtypes in rodent hepatocytes increases cellular lipid deposition (Ghosh et al., 2012). Moreover, liver sympathetic denervation reverses high‐fat diet (HFD)‐induced hepatic steatosis in mice (Hurr et al., 2019). In addition, we and others previously reported that β‐AR crosstalk with insulin signaling mediates AKT‐ and ERK‐dependent activation of the mTOR/S6K1 pathway in the heart (Ashraf et al., 2020; Morisco et al., 2005; Zhang et al., 2011). Therefore, chronic activation of β‐AR signaling during high‐calorie feeding may promote lipid deposition in the liver through mTORC1 hyperactivation.

In this study, we investigated the effect of acute β‐AR stimulation on the activity of liver ERK1/2, AKT, and mTOR/S6K1 signaling in mice under normal and high‐calorie feeding, and how changes in those pathways related to the amounts of triglyceride and glycogen stored in the tissue. Purified‐ingredient diets with varying amounts of fat and carbohydrate were used to determine the relative impact of both macronutrients on those parameters. Because fructose seems to exacerbate the lipogenic effect associated with high‐sugar intake (Ishimoto et al., 2012; Jensen et al., 2018), two isocaloric high‐sugar diets, a diet with mixed starch and simple sugars and a high‐sucrose diet (HSD), were included for direct comparison.

## 2 METHODS AND MATERIALS

### 2.1 Experimental animals

Eight‐week‐old male C57BL/6J mice (The Jackson Laboratory) were housed in the animal facilities of the Center for Comparative Research from the University of Mississippi Medical Center on a 12‐h light/12‐h dark cycle at a temperature of 22 ± 1°C and 40%–60% humidity. The study complied with the Guide for the Care and Use of Laboratory Animals: Eighth Edition (National Research Council, 2011) and was approved by the Institutional Animal Care and Use Committee of UMMC (protocol #1438A). All efforts were made to minimize animal suffering and to reduce the number of animals used.

### 2.2 Feeding and treatments

**FIGURE 1:**
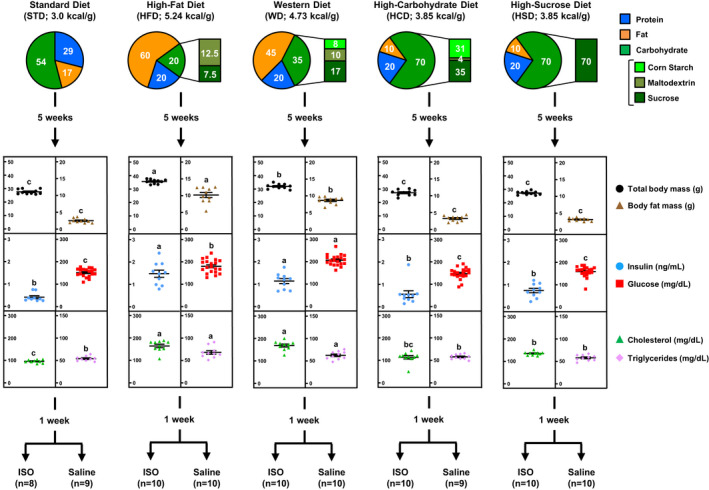
Flowchart of the experimental protocol depicting the impact of high‐calorie diet macronutrient composition on body and blood parameters. Eight‐week‐old male C57BL/6 mice (*n* = 99) were acclimated for 1 week on a nonpurified standard rodent chow diet (STD), after which mice were either kept on the same diet (*n* = 19) or randomly assigned with an Excel‐generated table to one of the following high‐calorie diets for another 6 week (*n* = 20 mice per diet): a high‐fat diet, a high‐fat/high‐sugar Western diet, a high‐sugar diet with mixed carbohydrates (HCD), and a high‐sucrose diet. After 5 week on the different diets, total body mass (*n* = 10), body composition (*n* = 10), and fasting values for blood glucose (*n* = 19–20), insulin (*n* = 9–10), total cholesterol (*n* = 9–10), and triglycerides (*n* = 9–10) were determined to assess the impact of the different high‐calorie diets on systemic metabolism. After a total of 6 week on the different diets, mice were subjected to a 5‐h fast prior to being randomly selected for an intraperitoneal injection with the beta‐adrenergic receptor agonist isoproterenol (ISO; 20 mg/kg) or saline as control. Saline and ISO treatments were performed in a nonlinear order and the experimentator was blinded to the type of injection. One hour after injection, mice were euthanized and liver tissue quickly collected for molecular and biochemical analyses. Three mice (two in the STD group and one in the HCD group) were excluded from posttreatment analyses due to health reasons. Data are expressed as mean ± SEM and were analyzed by one‐way ANOVA followed by Newman–Keuls test. Mean values without a common letter differ (*p* < 0.05)

The experimental protocol used in this study has been described previously (Ashraf et al., 2020). Briefly, all mice were fed ad libitum with a standard laboratory rodent diet (STD; Teklad 22/5 Rodent Diet no. 8640; Envigo) for 1 week, after which the animals were either maintained on the STD or randomly fed one of the following four high‐calorie diets (Figure 1): a HFD (Research Diets D12492; Research Diets), a high‐fat/high‐sugar Western diet (WD; Research Diets D12451), a high‐carbohydrate diet with mixed complex and simple sugars (HCD; Research Diets D12450B), or a HSD (Research Diets D07042201). Changes in body composition were assessed by EchoMRI (EchoMRI LLC). After 5 week on the different diets, mice were fasted for 5 h prior to determining glycemia with a handheld glucometer (Contour Next; Ascensia Diabetes Care). Next, the mice were briefly anesthetized with 5% isoflurane in order to draw blood in a heparinized tube via submandibular bleeding. Blood samples were centrifuged at 1500× g for 15 min at 4°C and plasma collected to determine circulating lipid levels with a Vet Axcel Clinical Chemistry System (Alfa Wassermann). After a total of 6 week on the different diets, mice were subjected to a final 5‐h fast starting at 08:00 prior to being injected intraperitoneally with 20 mg/kg of the β‐AR‐agonist isoproterenol (ISO) or saline as control. One hour after injection, mice were euthanized by cervical dislocation. Liver tissue was quickly excised, flash‐frozen in liquid nitrogen, and stored at −80°C until further analyses.

### 2.3 Western blotting

Frozen liver tissue samples were homogenized with a Bio‐Gen PRO200 Homogenizer (PRO Scientific) in protein lysis buffer containing 2.5 mmol/L ethylene glycol‐*bis*(2‐aminoethylether)‐*N*,*N*,*N'*,*N'*‐tetraacetic acid, 2.5 mmol/L EDTA, 20 mmol/L KCl, 40 mmol/L beta‐glycerophosphate, 40 mmol/L NaF, 4 mmol/L NaPPi, 10% (v/v) glycerol, 0.1% (v/v) Nonidet‐P40, complete protease inhibitor cocktail, and phosphatase inhibitor cocktails 2 and 3 (MilliporeSigma). Tissue homogenates were subsequently centrifuged at 16,000× g for 5 min at 4°C and protein concentration of the supernatant determined by bicinchoninic acid assay (Thermo Fisher Scientific). Proteins were separated by polyacrylamide gel electrophoresis in reducing sample buffer and transferred to 0.45 µm pore size polyvinylidene difluoride membranes. Membranes were blocked for 1 h with 5% (w/v) milk in 1× Tris‐buffered saline, 0.4% (v/v) Tween 20 at room temperature, and incubated overnight at 4°C with primary antibodies diluted in blocking solution. The phospho‐PKA substrate antibody was purchased from Cell Signaling Technology (Cat# 9624; RRID:AB_331817) and used at a 1:1500 dilution. All other primary antibodies used in this study have been previously reported (Ashraf et al., 2020). Protein detection was carried out on the following day using horseradish peroxidase‐conjugated secondary antibodies (Cell Signaling Technology) and chemiluminescent substrate (Thermo Fisher Scientific). Densitometry band quantifications were performed with ImageJ version 1.53g (National Institutes of Health; RRID:SCR_003070) and signals normalized to that of a “loading control” repeated on all membranes. In addition, the visualization of heat shock protein 60 (HSP60) was used to ascertain equal loading of samples between lanes. The amount of phosphorylation at each amino acid residue is expressed relative to the total amount of the protein investigated, which was detected on a separate set of membranes. Phospho‐PKA substrate levels are expressed relative to the total amount of proteins detected on membranes with Ponceau S staining.

### 2.4 Quantification of hepatic glycogen and triglycerides content

Glycogen was extracted by ethanol precipitation following 20 min digestion of tissue samples (~30 mg) in 30% w/v potassium hydroxide warmed at 70^°^C and quantified by enzymatic digestion with the method of Walaas and Walaas (1950). Triglycerides were extracted by homogenization of tissue samples (~60 mg) in 35% v/v heptane mixed with isopropanol and quantified with a colorimetric enzymatic assay (MyBioSource).

### 2.5 Statistical analyses

All data were statistically analyzed with the use of GraphPad Prism software version 9 (GraphPad Software; RRID:SCR_002798) and are expressed as means ± SEMs. Body and blood parameters presented on Figure 1 were analyzed by one‐way ANOVA followed by the Newman–Keuls post‐hoc test. All other data were analyzed by two‐way ANOVA followed by the Newman–Keuls post‐hoc test. For data with a nonsignificant interaction among the two factors, comparison among treatment means was performed as previously described (Wei et al., 2012). A *p* < 0.05 was considered statistically significant.

## 3 RESULTS

### 3.1 Development of metabolic syndrome with short‐term high‐calorie feeding depends on increased dietary fat intake, not on increased sugar consumption

The impact of the four different high‐calorie diets on male mouse body composition, blood metabolic parameters, glucose tolerance, and insulin sensitivity has been described in detail previously (Ashraf et al., 2020). In brief, compared to mice maintained on the STD, only mice fed the fat‐enriched diets HFD and WD experienced a greater increase in body weight gain that was linked to an increase in body fat mass (Figure 1). Both HFD and WD, but not HCD or HSD, were also associated with the development of glucose intolerance and insulin resistance (Ashraf et al., 2020). The HFD and WD also led to development of hyperinsulinemia, hyperglycemia, and dyslipidemia that was characterized by a more than 70% increase in total plasma cholesterol and a 16%–26% increase in plasma triglycerides (Figure 1). Although the HSD also increased total plasma cholesterol by 40%, all other metabolic parameters remained unchanged for mice fed with the sugar‐enriched diets HCD or HSD.

### 3.2 3‐phosphoinositide‐dependent protein kinase 1—But not mTORC2‐dependent phosphorylation of AKT is inhibited by sugar‐rich high‐calorie diets in liver of ISO‐treated mice

**FIGURE 2:**
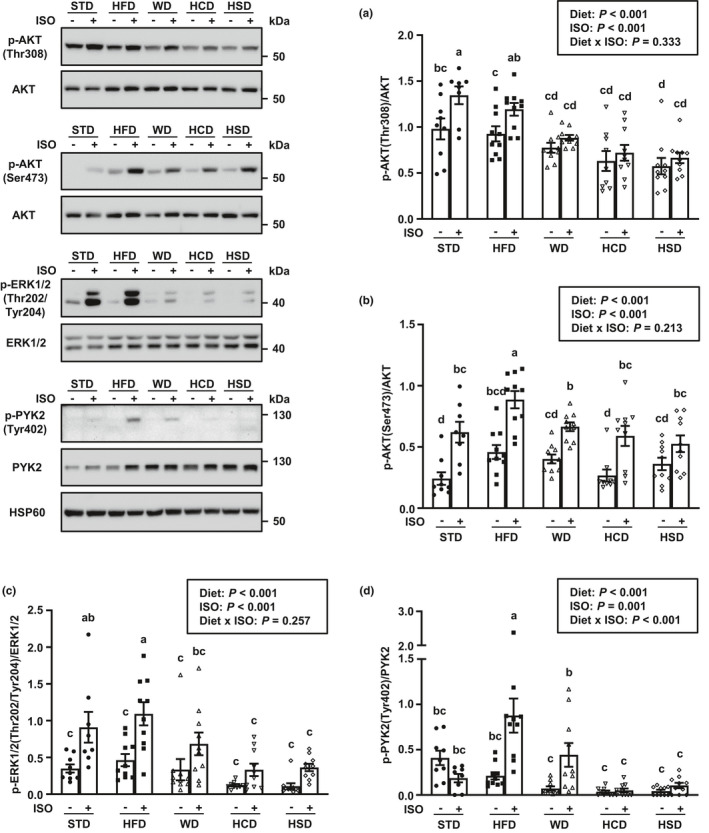
A high‐fat diet, but not high‐calorie diets with a high sugar content, is associated with increased signaling through AKT and ERK in liver of isoproterenol (ISO)‐treated mice. Phosphorylation levels of AKT threonine 308 residue (a), AKT serine 473 residue (b), ERK1/2 threonine 202 and tyrosine 204 residues (c), and PYK2 tyrosine 402 residue (d) in liver of male C57BL/6 mice fed a standard rodent chow diet (STD), a high‐fat diet (HFD), a high‐fat/high‐sugar Western diet (WD), a high‐sugar diet with mixed carbohydrates (HCD), or a high‐sucrose diet (HSD) for 6 week starting at 9 week of age and subsequently treated with ISO or saline for 1 h. HSP60 is shown as loading control. Data are expressed as mean ± SEM (*n* = 8–10 per group) and were analyzed by two‐way ANOVA followed by Newman–Keuls test. Mean values without a common letter differ (*p* < 0.05)

Full AKT activation depends on its phosphorylation at two sites; the threonine residue at position 308 (Thr308) which is located in the catalytic domain of the kinase and is phosphorylated by 3‐phosphoinositide‐dependent protein kinase 1 (PDK1) and serine 473 (Ser473) in the C‐terminal regulatory domain which is targeted by mTORC2 (Manning & Toker, 2017). Mice maintained on the STD throughout the study experienced an increase in AKT phosphorylation from baseline at both Thr308 and Ser473 residues with ISO stimulation (Figure 2a,b). While the amount of phosphorylation of AKT at Thr308 was similar to that of STD‐fed mice with HFD feeding, consumption of a HSD was associated with decreased Thr308 phosphorylation at baseline, and both of the high‐sugar HCD and HSD as well as the high‐fat and high‐sugar WD abrogated the increase in Thr308 phosphorylation in response to ISO treatment (Figure 2a). In stark contrast to these findings, basal and ISO‐stimulated phosphorylation of AKT at Ser473 remained unaffected by the consumption of WD, HCD, or HSD. In addition, consumption of a HFD potentiated AKT phosphorylation at Ser473 in response to ISO treatment (Figure 2b).

### 3.3 Protein tyrosine kinase 2 beta activation is associated with preserved ISO‐mediated ERK stimulation in liver of mice fed high‐calorie diets with a high‐fat content

The activity of the protein tyrosine kinase 2 beta (PYK2), a known upstream activator of ERK signaling pathway, is reportedly increased in hepatocytes upon increased glucose or fructose supply (Bandyopadhyay et al., 2000; Fernandez‐Novell et al., 2014). Activation of PYK2 by phosphorylation of its tyrosine residue at position 402 (Tyr402) is also highly susceptible to β‐AR stimulation (Chruscinski et al., 2013). In the STD‐fed mice, phosphorylation of ERK1/2 at the activating sites threonine 202 and tyrosine 204 (Thr202/Tyr204), but not PYK2 phosphorylation at Tyr402, increased upon ISO treatment (Figure 2c,d). Similar to AKT phosphorylation at Thr308, the stimulatory effect of ISO on Thr202/Tyr204 phosphorylation was completely lost with the high‐sugar diets HCD and HSD. However, ISO‐mediated phosphorylation of ERK1/2 was preserved with consumption of a HFD or WD (Figure 2c). Interestingly, preservation of the β‐AR signaling‐mediated increase in ERK1/2 activity with the fat‐enriched, high‐calorie diets was associated with an enhanced phosphorylative activation of PYK2 at Tyr402 (Figure 2d).

### 3.4 AKT‐ and ERK‐dependent inhibition of Tuberous sclerosis complex 2 is enhanced by high‐fat feeding in liver of mice treated with ISO

**FIGURE 3:**
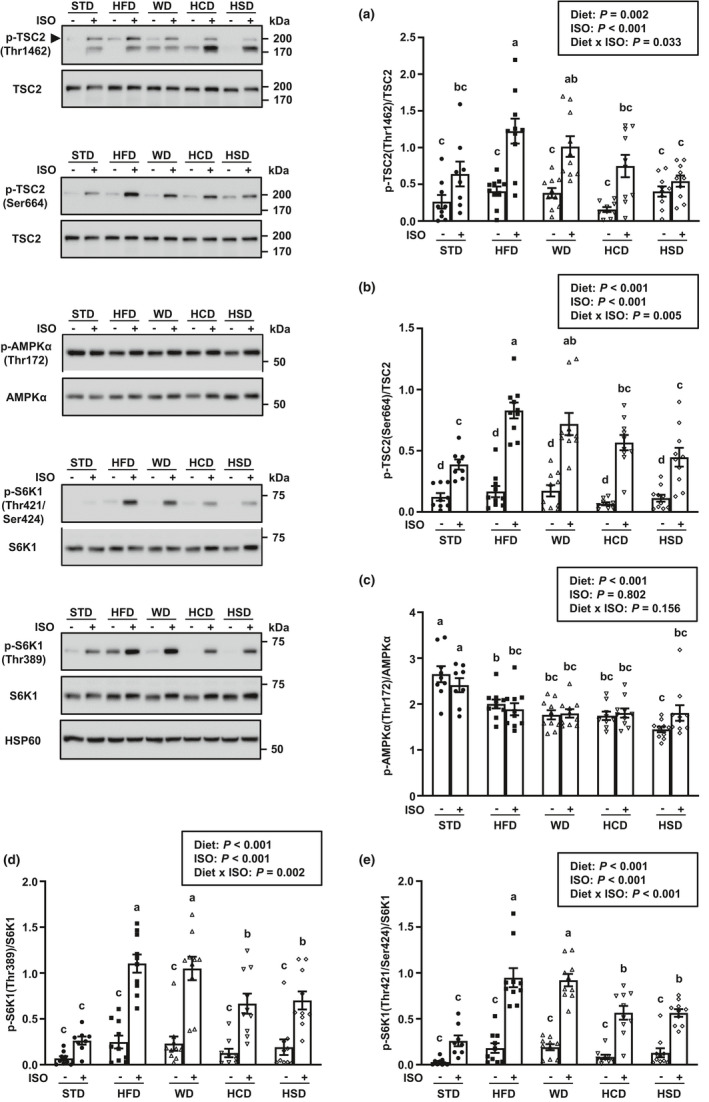
Increased inhibitory phosphorylation of TCS2 by ERK and AKT and decreased activity of AMP‐activated protein kinase (AMPK) favor mTORC1/S6K1 hyperactivation in liver of high‐fat diet‐fed mice treated with ISO. Phosphorylation levels of TSC2 threonine 1462 residue (a), TSC2 serine 664 residue (b), AMPK α subunit threonine 172 residue (c), S6K1 threonine 389 residue (d), and S6K1 threonine 421 and serine 424 residues (e) in liver of male C57BL/6 mice fed a standard rodent chow diet (STD), a high‐fat diet (HFD), a high‐fat/high‐sugar Western diet (WD), a high‐sugar diet with mixed carbohydrates (HCD), or a high‐sucrose diet (HSD) for 6 week starting at 9 week of age and subsequently treated with isoproterenol (ISO) or saline for 1 h. HSP60 is shown as loading control. Data are expressed as mean ± SEM (*n* = 8–10 per group) and were analyzed by two‐way ANOVA followed by Newman–Keuls test. Mean values without a common letter differ (*p* < 0.05). S6K1, ribosomal protein S6 kinase beta‐1

Tuberous sclerosis complex 2, also known as tuberin, acts as a critical negative regulator of mTORC1. The phosphorylation of TSC2 by various molecular signaling pathways serves as an integration point for the regulation of metabolism and cell growth by environmental signals (Saxton & Sabatini, 2017). Primary points of control for the stimulation of mTORC1 by AKT and ERK include phosphorylation of TSC2 at amino acid residues threonine 1462 (Thr1462) and serine 664 (Ser664), respectively (Inoki et al., 2002; Ma et al., 2005). In consistence with the enhanced activity of AKT and ERK, TSC2 phosphorylation at both Thr1462 and Ser664 increased for STD‐fed mice subjected to the ISO treatment (Figure 3a,b). Although none of the high‐calorie diets impacted TSC2 regulation by AKT under baseline conditions, HFD amplified, while HSD abrogated, phosphorylation of TSC2 at Thr1462 under β‐AR stimulation (Figure 3a). Likewise, TSC2 inhibition by ERK was unaltered with high‐calorie feeding at baseline but was enhanced with consumption of the fat‐enriched HFD and WD during ISO treatment (Figure 3b).

Conversely to the AKT and ERK permissive actions on mTORC1 signaling, AMPK leads to diminished mTOR activity through stabilization of the TSC1–TSC2 complex and through direct inhibitory phosphorylation of Raptor in the mTORC1 complex (Gwinn et al., 2008; Inoki et al., 2003). However, activation of the catalytic (alpha) subunit of AMPK through phosphorylation of threonine residue at position 172 (thr172) was unaffected by β‐AR stimulation, and all high‐calorie diets led to a similar decrease in AMPK activity when compared to STD‐fed mice (Figure 3c). Therefore, β‐AR‐mediated activation of mTORC1 in liver was likely intensified with consumption of high‐calorie diets with a high‐fat content.

### 3.5 High dietary fat content exacerbates the increase in ERK‐ and mTORC1‐dependent activation of S6K1 mediated by high‐calorie feeding in liver of mice treated with ISO

In spite of the noticeable increases in AKT and ERK activation resulting in greater TSC2 inhibition, ERK‐ and mTORC1‐dependent phosphorylation of S6K1 failed to increase significantly in STD‐fed mice acutely treated with ISO (Figure 3d,e). Consistent with the unchanged phosphorylation levels of those same upstream regulators under baseline conditions, phosphorylation of S6K1 similarly remained unaffected by high‐calorie feeding alone. However, β‐AR stimulation induced a dramatic increase in S6K1 stimulatory phosphorylation at both ERK‐dependent (Thr421/Ser424) and mTORC1‐dependent (Thr389) sites with all four high‐calorie diets. Interestingly, activation of S6K1 at both sites was 50% to 68% greater with the fat‐enriched HFD and WD when compared to high‐sugar HCD and HSD (Figure 3d,e).

### 3.6 Beta‐adrenergic receptor stimulation promotes hepatic glycogen breakdown in mice fed a high‐sugar HCD or HSD, but not in mice on a fat‐enriched HFD or WD

**FIGURE 4:**
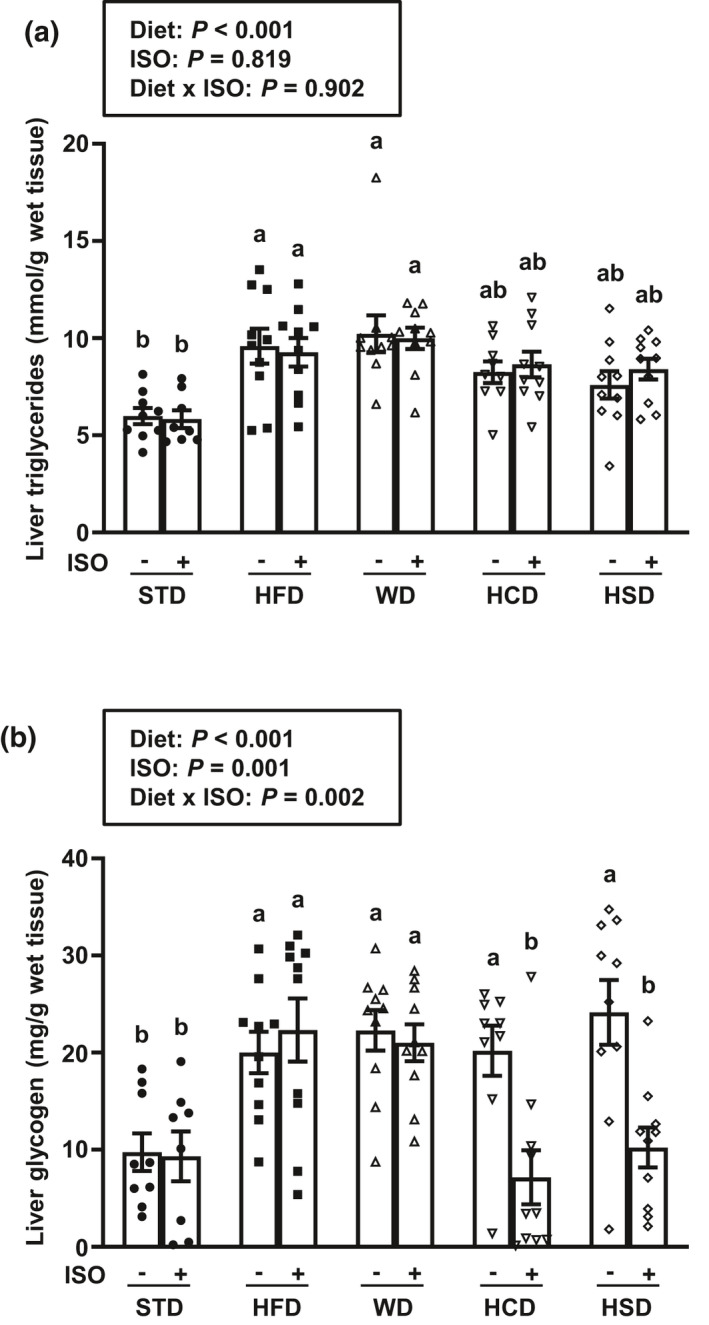
Dietary fat, but not sugar, inhibits β‐adrenergic receptor‐mediated glycogenolysis in liver of high‐calorie diet‐fed mice. (a) Triglyceride levels and (b) glycogen content in liver of male C57BL/6 mice fed a standard rodent chow diet (STD), a high‐fat diet (HFD), a high‐fat/high‐sugar Western diet (WD), a high‐sugar diet with mixed carbohydrates (HCD), or a high‐sucrose diet (HSD) for 6 week starting at 9 week of age and subsequently treated with isoproterenol (ISO) or saline for 1 h. Data are expressed as mean ± SEM (*n* = 8–10 per group) and were analyzed by two‐way ANOVA followed by Newman–Keuls test. Mean values without a common letter differ (*p* < 0.05)

Acute β‐AR stimulation had no impact on fasting hepatic triglyceride and glycogen contents for mice maintained on the STD (Figure 4a,b). Compared to STD, both HFD and WD increased baseline hepatic triglyceride content more than 1.6‐fold, while HCD and HSD led to a more moderate (~1.3‐fold) increase. Acute ISO treatment also had no impact on hepatic triglyceride content in high‐calorie diet‐fed mice (Figure 4a). Regardless of their macronutrient composition, all four high‐calorie diets increased fasting hepatic glycogen content more than twofold. Interestingly, the ISO treatment decreased glycogen stores back to normal fasting levels for mice fed a high‐sugar HCD or HSD, an effect that was blocked in animals on a fat‐enriched HFD or WD (Figure 4b).

### 3.7 ISO‐mediated inhibition of glycogen synthesis is enhanced by sugar‐rich high‐calorie diets

**FIGURE 5:**
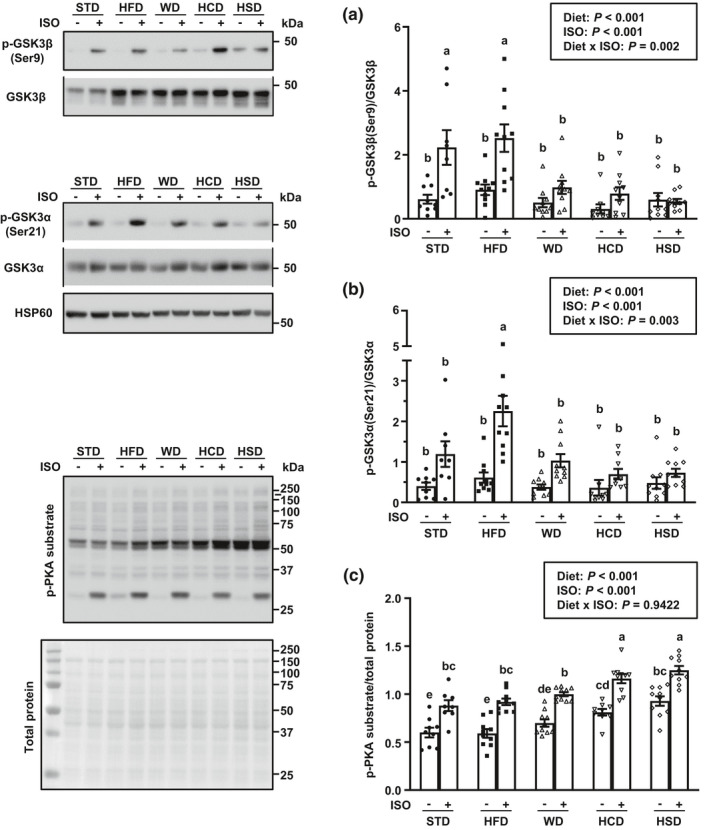
High‐calorie diets with a high sugar content promote inhibition of glycogen synthesis during isoproterenol (ISO) stimulation in mouse liver. (a) Phosphorylation levels of GSK3β at serine 9 residue, (b) phosphorylation levels of GSK3α at serine 21 residue, and (c) phosphorylation of PKA protein targets in liver of male C57BL/6 mice fed a standard rodent chow diet (STD), a high‐fat diet (HFD), a high‐fat/high‐sugar Western diet (WD), a high‐sugar diet with mixed carbohydrates (HCD), or a high‐sucrose diet (HSD) for 6 week starting at 9 week of age and subsequently treated with ISO or saline for 1 h. HSP60 is shown as loading control. Data are expressed as mean ± SEM (*n* = 8–10 per group) and were analyzed by two‐way ANOVA followed by Newman–Keuls test. Mean values without a common letter differ (*p* < 0.05)

In addition to stimulation of glycogenolysis, increased glycogen breakdown may occur through inhibition of glycogen synthesis. Besides its allosteric regulation by glucose‐6‐phosphate, the enzyme glycogen synthase can be inactivated through direct phosphorylation by AMPK, glycogen synthase kinase‐3 (GSK3), and increased cAMP/PKA signaling (Feher, 2017; Nielsen & Richter, 2003). As mentioned previously, liver AMPK activity was unresponsive to ISO stimulation and decreased similarly with all four high‐calorie diets (Figure 3c). Inhibition of GSK3β by AKT followed the phosphorylation pattern of AKT at Thr308: Phosphorylation at serine 9 (Ser9) was abrogated with HCD, HSD, and WD but remained intact with HFD feeding (Figure 5a). Consumption of HFD also led to enhanced inhibitory phosphorylation of GSK3α at the serine 21 (Ser21) residue (Figure 5b). In addition, basal PKA activity was increased with consumption of the high‐sugar diets and further enhanced during ISO stimulation (Figure 5c). Thus, inhibition of hepatic glycogen synthesis may be stronger with sugar‐rich, high‐calorie diets due to a combined absence of GSK3 inhibitory phosphorylation by AKT and to an augmentation of cAMP/PKA signaling under ISO stimulation.

## 4 DISCUSSION

While an essential role for mTOR in the regulation of liver metabolism is now widely recognized, our understanding of the upstream signals modulating this pathway and of their involvement in the pathogeneses of the metabolic syndrome and NAFLD remains incomplete (Bar‐Tana, 2020; Lamming & Sabatini, 2013). In the present study, we demonstrate that β‐AR signaling 1‐ promotes AKT‐ and ERK1/2‐mediated inhibition of TSC2 in mouse liver and 2‐ leads to the hyperactivation of mTORC1 and S6K1 in response to excess dietary fat and/or sugar supply. We also provide evidence that the interaction of β‐AR signaling with excess nutrients supply alters metabolism and hepatic fuel storage. Altogether, the findings may provide a molecular explanation as to why a chronic increase in hepatic β‐AR activity has been linked to diet‐induced hepatic lipid accumulation in rodents (Ghosh et al., 2012; Hurr et al., 2019).

The first significant finding of this study was the demonstration that in vivo stimulation of β‐AR signaling with the nonselective agonist ISO increased AKT‐ and ERK‐dependent activation of mTORC1 in the liver of mice fed a STD. Although it has been known for some time that β‐AR signaling can regulate the insulin transduction machinery in cardiomyocytes through PKA/Ca^2+^‐and phosphoinositide 3‐kinase‐dependent phosphorylation of AKT, our knowledge of the extent of the crosstalk between both pathways and of its involvement in the insulin‐independent stimulation of S6K1 is more recent (Ashraf et al., 2020; Morisco et al., 2005; Zhang et al., 2011). Interestingly, Zhang et al. initially reported that the β‐AR/insulin signaling crosstalk mechanism is restricted to the heart and does not occur in other tissues including lung, kidney, or liver (Zhang et al., 2011). Our results are clearly in contrast to their findings. Although liver S6K1 phosphorylation was only modestly increased under STD feeding, the >two‐fold increase in AKT‐ and ERK‐mediated inhibitory phosphorylation of TSC2 suggests a significant activation of mTORC1 with ISO stimulation. Differences between the experimental protocols used in the two studies and particularly changes in the macronutrient composition of the rodent diets employed may have contributed to this discrepancy.

**FIGURE 6:**
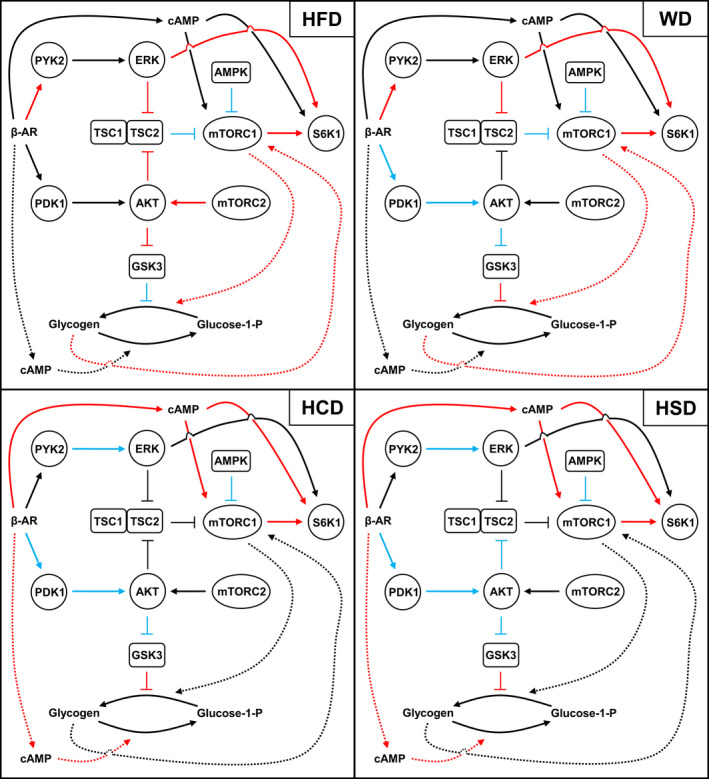
Proposed mechanisms linking high dietary intake of fat and sugar to loss of hepatic metabolic homeostasis under beta‐adrenergic receptor (β‐AR) stimulation. Pathways drawn in red indicate increased activity, whereas pathways drawn in blue denote decreased activity, compared to mice maintained on a standard rodent chow diet (STD). Dotted lines and arrows represent pathways with a proposed involvement in the observed effects that were not directly investigated in the present study. HCD, high‐sugar diet with mixed carbohydrates; HFD, high‐fat diet; HSD, high‐sucrose diet; WD, high‐fat/high‐sugar Western diet

Because of the reliance of mammalian cells on amino acids, glucose, and lipids for growth and division, mTOR has evolved as a universal sensor for all three types of dietary macronutrients (Foster, 2013; Yuan et al., 2013). Although we cannot exclude a role for subtle changes in dietary amino acid composition in mediating the molecular signaling differences between STD and the other diets, all four high‐calorie diets have matched formulas and were composed of purified ingredients. Therefore, variations in the phosphorylation levels of liver AKT, ERK1/2, and S6K1 between the high‐calorie diets can be traced back to changes in the relative amounts of dietary fat and sugar. Hence, our results indicate that high dietary fat is an important contributing factor to the hyperactivation of hepatic mTORC1 mediated by ERK and AKT under β‐AR stimulation. Interestingly, the fat‐enriched HFD and WD were also the only two high‐calorie diets associated with marked development of metabolic syndrome features, namely hyperinsulinemia, hyperglycemia, and hyperlipidemia (Figure 1). Considering the central role of hyperinsulinemia and hyperglycemia in the development of selective hepatic insulin resistance and in the activation of AKT, ERK1/2, and PYK2 in hepatocytes (Brown & Goldstein, 2008; Fernandez‐Novell et al., 2014), it is conceivable that both factors contributed to the greater stimulation of the kinases in response to β‐AR stimulation. However, the fact that the essentially sugar‐enriched HCD and HSD still led to exacerbation of S6K1 phosphorylation while failing to amplify PYK2/ERK1/2 signaling, and simultaneously leading to the abrogation of AKT phosphorylation by PDK1, suggests that additional molecular pathways independent from AKT and ERK1/2 are implicated in β‐AR‐mediated hyperactivation of the mTORC1/S6K1 axis in response to high‐calorie feeding (Figure 6).

Activation of the energy sensor AMPK leads to the inhibitory phosphorylation of mTORC1 (Yuan et al., 2013). In keeping with the notion that excess nutrients supply suppresses AMPK signaling (Coughlan et al., 2013), decreased phosphorylation of the AMPK subunit α at Thr172 may have contributed to the enhancement of hepatic mTORC1 signaling in response to ISO treatment for all four high‐calorie diets. Considering that chronic exposure to an obesogenic diet has been reported to upregulate hepatic PKA signaling in mice, greater activation of the canonical β‐AR signaling with high‐calorie feeding is another likely candidate pathway for mTORC1 hyperactivation (London et al., 2017). Indeed, the cAMP/PKA‐dependent activation of S6K1 has been well documented in various system models (Brewer et al., 2007; Cass & Meinkoth, 1998; Coulonval et al., 2000; Dremier et al., 2007; Suh et al., 2003). Mechanistically, it has been reported that cAMP activates mTORC1 by shifting the binding and interaction of Rheb from cAMP phosphodiesterase 4D to mTOR (Kim et al., 2010). Importantly, phosphorylation of S6K1 at Thr389 by mTORC1 is facilitated by prior phosphorylation of the amino acid residues Thr421/Ser424 in the autoinhibitory domain of the kinase, the latter being partly induced by a cAMP‐dependent mechanism in certain conditions (Blancquaert et al., 2010; Dennis et al., 1998). Interestingly, we found that PKA‐mediated protein phosphorylation was enhanced with consumption of HCD and HSD (Figure 5c). Therefore, we postulate that both decreased activity of AMPK and increased stimulation of the cAMP/PKA axis account for most of the enhancement in mTORC1/S6K1 signaling and increased glycogenolysis with the high‐sugar HCD and HSD (Figure 6). In addition, since the consumption of HCD and HSD yielded similar results, this indicates that the greater lipogenic properties of fructose do not depend on the investigated mechanisms.

In terms of hepatic lipid storage, consumption of the fat‐enriched HFD and WD led to greater triglyceride accumulation, which is consistent with previous findings that dietary fat intake in mice promotes the development of hepatic steatosis (Meijer et al., 2010). While this does not establish proof of causation, it is noteworthy that increased triglyceride storage positively correlated with the greater capacity for HFDs to stimulate S6K1 under ISO treatment. A 24‐h‐long treatment of isolated hepatocytes with ISO has been reported to stimulate lipolysis and to decrease the intracellular pool of stored triglycerides (Schott et al., 2017). However, acute in vivo β‐AR stimulation was not presently associated with any change in liver triglyceride content in the STD‐ or high‐calorie diet‐fed mice. Interestingly, the fat‐enriched HFD and WD were also associated with an inhibition of β‐AR‐mediated mobilization of the excess fasting glycogen stores caused by high‐calorie feeding. Both hyperinsulinemia and hyperglycemia have been implicated in the reciprocal regulation of glycogen metabolism, with hyperinsulinemia inhibiting net hepatic glycogenolysis primarily through inhibition of glycogen synthase flux and hyperglycemia acting in parallel through inhibition of glycogen phosphorylase flux (Petersen et al., 1998). While still underappreciated, the stimulation of glycogen synthesis by mTORC1 through SREBP‐mediated regulation of protein targeting to glycogen represents another important crosstalk mechanism in the control of liver metabolism (Lu et al., 2014), and we propose that this mechanism may also be enhanced during high‐fat feeding in response to β‐AR stimulation to serve as a feedback system meant to inhibit glycogenolysis in order to prevent the exacerbation of hyperglycemia. The existence of another positive feedback loop in which excess glycogen accumulation, in turn, stimulates the mTORC1/SREBP1 pathway to shift energy storage to lipogenesis, may also explain why HFD and WD were associated with greater lipid accumulation (Figure 6; Lu et al., 2014).

Further investigations are needed to clarify the signaling mechanisms that led to our observations. First of all, it remains unclear whether the differential changes in molecular pathway activities were caused directly by changes in the macronutrient composition of the diets, or indirectly through variations in body weight gain and adiposity. In relation to this point, we cannot ascertain that the differential modulation of liver AKT, ERK1/2, PKA, and mTORC1/S6K1 kinases was entirely dependent on local β‐AR signaling. Indeed, β‐adrenergic stimulation has long been known to augment basal insulin and glucagon secretion (Gerich et al., 1974; Porte, 1967). Increased glucagon secretion is fundamental to the activation of hepatic cAMP/PKA signaling and the regulation of glycogen metabolism (Han et al., 2016). Neither did we determine the consequences of macronutrient composition for fasting glucagon levels nor how insulin and glucagon secretions were impacted by ISO under the different feeding conditions. Glucagon‐like peptide 1 (GLP‐1) may also be secreted in vivo in response to ISO treatment (Hansen et al., 2004), and although GLP‐1 signaling rather decreases steatosis by promoting fat oxidation, stimulation of the GLP‐1 receptor on hepatocytes has also been associated with increased activation of AKT and ERK1/2 (Aviv et al., 2009; Gupta et al., 2010; Svegliati‐Baroni et al., 2011) in those cells. Another limitation of our study is that our investigations were performed at the conventional laboratory rodent housing temperature of 22°C, which is likely to have impacted the metabolic response of the mice to the dietary protocol and ISO treatment (Reitman, 2018). Lastly, the relatively short duration of the ISO treatment was not designed to investigate a potential link between long‐term β‐AR‐mediated stimulation of the mTORC1/S6K1 pathway and increased hepatic lipid accumulation. While the determination of the impact of chronic β‐AR stimulation on HFD‐induced NAFLD was recently attempted by others with continuous ISO infusion but yielded negative results (Nakade et al., 2020), it should be kept in mind that intrinsic biological system parameters such as signal frequency rather than the amplitude of stimulations often play a critical role in the physiological and metabolic responses to neuroendocrine and endocrine signals (Gan & Quinton, 2010; Paolisso et al., 1991).

In conclusions, our study has identified β‐AR signaling as a novel positive regulator of mTORC1 in mouse liver. In addition, our findings demonstrate that excess dietary intake of fat and sugar leads to hyperactivation of the hepatic mTORC1/S6K1 axis following β‐AR stimulation. The exacerbation of S6K1 activation was greater with fat‐enriched diets due to molecular crosstalk mechanisms implicating the recruitment of insulin‐ and glucose‐regulated AKT and PYK2/ERK1/2 pathways, whereas high‐sugar diets seemed to rely principally on other mechanisms including suppression of AMPK signaling and activation of cAMP‐dependent pathways. At the metabolic level, fat‐enriched, high‐calorie diets contributed to more triglyceride accumulation that was linked to an excess glycogen storage resistant to β‐AR‐mediated breakdown. These findings provide further rationale for the development of therapies aimed at targeting augmented β‐AR signaling in the pathogenesis of NAFLD (Ghosh et al., 2012; Hurr et al., 2019).

## DISCLOSURES

No conflicts of interest, financial or otherwise, are declared by the authors.

## AUTHOR CONTRIBUTIONS

Sadia Ashraf and Romain Harmancey conceived and designed research; Sadia Ashraf, Gizem Yilmaz, and Romain Harmancey performed experiments; Sadia Ashraf, Nadia Ashraf, Gizem Yilmaz, and Romain Harmancey analyzed data; Sadia Ashraf, Nadia Ashraf, and Romain Harmancey interpreted results of the experiments; Sadia Ashraf, Nadia Ashraf, Gizem Yilmaz, and Romain Harmancey prepared figures; Sadia Ashraf and Romain Harmancey drafted manuscript; Sadia Ashraf, Nadia Ashraf, and Romain Harmancey edited and revised manuscript; All authors approved final version of the manuscript.

## Data Availability

The data that support the findings of this study are available from the corresponding author upon reasonable request from a qualified researcher.
